# Molecular modeling of antibodies for the treatment of TNF *α*‐related immunological diseases

**DOI:** 10.1002/prp2.197

**Published:** 2016-01-15

**Authors:** Ciro Leonardo Pierri, Fabrizio Bossis, Giuseppe Punzi, Anna De Grassi, Michela Cetrone, Giovanni Parisi, Domenico Tricarico

**Affiliations:** ^1^ Department of Biosciences, Biotechnologies and BiopharmaceuticsUniversity of BariBariItaly; ^2^ Pharmacovigilance unitRegione PugliaASL BABariItaly; ^3^ Department of Pharmacy – Drug ScienceUniversity of BariBariItaly

**Keywords:** TNFalpha modeling, antibody‐receptor docking, antibody‐receptor complex stability, anti‐TNF alpha antibody biosimilar, Fc glycosylation

## Abstract

Therapeutic monoclonal antibodies (mAbs) have high efficacy in treating TNF *α*‐related immunological diseases. Other than neutralizing TNF *α*, these IgG1 antibodies exert Fc receptor‐mediated effector functions such as the complement‐dependent cytotoxicity (CDC) and antibody‐dependent cell cytotoxicity (ADCC). The crystallizable fragment (Fc) of these IgG1 contains a single glycosylation site at Asn 297/300 that is essential for the CDC and ADCC. Glycosylated antibodies lacking core fucosylation showed an improved ADCC. However, no structural data are available concerning the ligand‐binding interaction of these mAbs used in TNF *α*‐related diseases and the role of the fucosylation. We therefore used comparative modeling for generating complete 3D mAb models that include the antigen‐binding fragment (Fab) portions of infliximab, complexed with TNF *α* (4G3Y.pdb), the Fc region of the human IGHG1 fucosylated (3SGJ) and afucosylated (3SGK) complexed with the Fc receptor subtype Fc*γ* RIIIA, and the Fc region of a murine immunoglobulin (1IGT). After few thousand steps of energy minimization on the resulting 3D mAb models, minimized final models were used to quantify interactions occurring between Fc*γ* RIIIA and the fucosylated/afucosylated Fc fragments. While fucosylation does not affect Fab‐TNF *α* interactions, we found that in the absence of fucosylation the Fc–mAb domain and Fc*γ* RIIIA are closer and new strong interactions are established between G129 of the receptor and S301 of the *Chimera 2* Fc mAb; new polar interactions are also established between the *Chimera 2* Fc residues Y299, N300, and S301 and the Fc*γ* RIIIA residues K128, G129, R130, and R155. These data help to explain the reduced ADCC observed in the fucosylated mAbs suggesting the specific AA residues involved in binding interactions.

**Abbreviations**
ADCC: antibody‐dependent cell cytotoxicity
AR: rheumatoid arthritis
AS: ankylosing spondylitis
BMA: *β*‐d‐mannose
C1q: complement component 1, subcomponent q
CDC: complement‐dependent cytotoxicity
CD: Chron's disease
EMA: European medicinal agency
Fab: antigen‐binding fragment
Fc: crystallizable fragment
Fc*γ*RIIIA: Fc gamma receptor III A
FDA: Food and Drug Administration
FUC: *α*‐l‐fucose
GAL: *α*‐d‐galactose
IBD: inflammatory bowel disease
mAbs: monoclonal antibodies
MAN: *α*‐d‐mannose
NAG: *N*‐acetyl‐glucosamine
PsA: psoriasis arthritis
TNFR: tumor necrosis factor receptor
TNF*α*: tumor necrosis factor alpha
UC: ulcerative colitis

## Introduction

Therapeutic monoclonal antibodies (mAbs) targeting tumor necrosis factor alpha (TNF*α*) have high efficacy in treating TNF*α*‐related immunological diseases such as ankylosing spondylitis (AS), rheumatoid arthritis (AR), and skin diseases as psoriasis arthritis (PSA), as well as Chron's disease (CD) and ulcers colitis (UC) affecting gastrointestinal apparatus. Several mAbs are currently available, such as infliximab, etanercept, adalimumab, certolizumab pegol, and golimumab. The biosimilars of infliximab Inflectra^®^ (Hospira UK Limited, Queensway, Royal Leamington Spa, Warwickshire CV31 3RW, UK) and Remsima^®^ (Celltrion Healthcare Hungary Kft. 1023 Budapest, Á rpád Fejedelem útja 26‐28) have been recently approved following an extensive comparative exercise evaluation as determined by European Medicinal Agency (EMA), Food and Drug Administration (FDA), and Health Canada (EMAIAR, 2013; EMARAR, 2013).

The primary mechanism of action of these mAbs involves the binding of the antigen‐binding fragment (Fab) of the IgG1 to the circulating and membrane bound TNF*α* antigen. The sites of interaction and the AA residues responsible for the binding of the Fab fragment to the antigen were recently reported for the first time for infliximab (Liang et al. 2013). Crystallography revealed that there is only one TNF*α*/infliximab–Fab complex in one asymmetric unit. The structure revealed a 3:3 molar ratio of TNF*α* and infliximab–Fab complex. The E‐F loop is critical in determining the high affinity and specificity of the binding of infliximab to TNF*α* versus TNF*β* (Liang et al. 2013).

The reported binding of infliximab to the soluble antigen neutralizes its actions in the treated tissues. Indeed, the binding affinity of infliximab–Fab to soluble TNF*α* is in the picomolar concentration range and the avidity to the membrane‐associated TNF*α* is in the nanomolar concentration and is comparable with the capability of the tumor necrosis factor receptor (TNFR1) subtype to bind the ligand TNF*α* (Kaymakcalan et al. 2009). Furthermore, infliximab prevents the TNF*α*–TNFR interaction by occupying the same binding site of TNF*α*. The binding of infliximab to the TNF*α* may additionally prevent the TNF*α* receptor activation and mediate programmed cell death in a variety of cell types leading to apoptosis.

Despite the deep knowledge of TNF*α*–infliximab–Fab interacting residues, very little is known about interactions between the anti‐TNF*α* mAbs and the crystallizable fragment (Fc) receptors. Nevertheless, mAbs–Fc receptor interactions are crucial for the activation of the immune response (Nimmerjahn and Ravetch 2008). The complement component C1q (complement component 1, subcomponent q) can bind to the Fc portion of antibodies and cause the activation of a cascade resulting in complement‐dependent cytotoxicity (CDC). The Fc region of the antibody can also interact with the Fc receptors subtypes such as the Fc*γ*RIIIA (CD16) receptor on natural killer (NK) cells as well as on other myeloid cells, inducing the release of cytokines such as IFNgamma and cytotoxic perforin and granzymes, which culminates in antibody‐dependent cell cytotoxicity (ADCC) (Kim et al. 2006; Geremia et al. 2014). The NK cell type is for instance involved in the innate immune response and its proliferation is also finely regulated by second messengers and voltage‐dependent K^+^ ion channels which are known to regulate cell proliferation in different cell types (Koshy et al. 2013; Curci et al. 2014). The ADCC is an important biological function attributed to the mechanism of action of several therapeutic antibodies, in particular for oncology targeting mAbs (Parekh et al. 2012).

The level of glycosylation of the IgG1 antibodies at the Fc domain plays a crucial role in triggering CDC and ADCC mechanisms. Fc glycosylation of human IgGs occurs in a conserved *N*‐glycosylation site within the CH2 domain, where glycans are linked to the Asn297/300. It is also known that antibodies lacking glycosylation residues at this position show an impaired binding of IgG1 to Fc receptors and C1q with absent ADCC and CDC activity, respectively (Zhou et al. 2008). The resulting complex consists of a biantennary oligosaccharide containing two *N*‐acetylglucosamine (GlcNAc) and three mannose, two of which are linked to two terminal GlcNAc (IgG1–G0). Two additional galactose (IgG1–G1) or galactose plus sialic acid (IgG1–G2) linked to the two terminal GlcNAc can be present in some glycosylated IgG1 variants. Furthermore, a fucose (IgG1–G0F/G1F) residue can be also observed linked to the first GlcNAc of the structure. The level of fucosylation in the Fc fragment is determined by the enzyme fucosyl transferase expressed in the cell lines used for bioproduction. Both cell lines deleted for the fucosyl transferase gene and cell lines treated with fucosyl transferase inhibitors express IgG1 afucosylated or poorly fucosylated (Zhou et al. 2008; Ferrara et al. 2011).

It is known that glycosylated mAbs with an additional fucose residue may show reduced or absent secondary pharmacodynamic actions associated with the ADCC mechanism; conversely glycosylated mAbs lacking core fucosylation show an improved ADCC and/or CDC (Davies et al. 2001; Shields et al. 2002; Shinkawa et al. 2003; Kapur et al. 2014). For example, the anti‐CD20 mAbs ofatumumab and rituximab display different levels of B‐cell depletion due to altered fucosylation levels. The recently developed biosimilar of infliximab (Inflectra^®^ and Remsima^®^) failed to induce ADCC in the NK cells possibly due to elevated fucosylation of the Fc fragment (EMAIAR, 2013; EMARAR, 2013). Whether the loss of ADCC mechanism associated to fucosylation is related to cellular factors affecting mAb binding to the antigen or to impaired ligand‐binding interactions involving specific AA residues is not known.

Although the crystal structures of three entire mAbs, that is, 1IGT (Harris et al. 1997), 1IGY (Harris et al. 1998), and 1HZH (Saphire et al. 2001) and of several mAb fragments in complex with their interactors, that is, a human IgG1–Fc fragment in complex with its receptor, Fc*γ*RIII (see 3SGJ, 3SGK) (Sondermann et al. 2000), and the infliximab Fab fragment in complex with TNF*α* (see 4G3Y) (Liang et al. 2013) are available on the protein data bank, the influence of the Fc fucosylation on the molecular interactions between mAbs and Fc receptors have never been investigated.

In the present work we provided for the first time three complete mAb models that included the Fab portions of infliximab complexed with TNF*α* (coordinates from 4G3Y), the Fc region of a human IGHG1 fucosylated (3SGJ) or afucosylated (3SGK) complexed with Fc*γ*RIIIA, and the Fc portion of a murine immunoglobulin (1IGT).

The superimposition of the 3D mAb models allowed us to calculate and evaluate the number and quality of interactions at the interface between Fc*γ*RIIIA glycans and the fucosylated/afucosylated Fc in the simultaneous presence of the IgG1, the TNF*α*, and the Fc receptor.

We observed that while fucosylation appears to not affect Fab–TNF*α* interactions, the superimposition of the 3D mAb‐generated chimeras revealed a change in the number and quality of interactions at the interface between Fc*γ*RIIIA and the Fc fragments in the presence or absence of fucose. In the absence of fucosylation, the Fc and Fc*γ*RIIIA are quite closer and in particular new strong interactions are established between G129 of the receptor Fc*γ*RIIIA and S301 of the Fc fragment of the mAb as well as new polar interactions are established between Y299, N300, and S301 from the Fc fragment of the mAb and residues K128, G129, R130, and R155 from the Fc*γ*RIIIA receptor. The obtained in silico data help to explain the reduced ADCC observed in vitro in the fucosylated therapeutic mAbs indicating the specific AA residues involved in ligand‐binding interactions. This work provides a structural basis for evaluating drug–protein interactions of anti‐TNF*α* mAbs to improve the Fc mAbs affinity with the Fc receptor and ADCC activity in the immunological diseases.

## Materials and Methods

### Sequence alignment

We used ClustalW implemented in Jalview for aligning the sequences of the Fab fragments and the sequences of the Fc portions extracted from a murine immunoglobulin (PDB_ID: 1IGT), from the infliximab (PDB_ID: 4G3Y), and from a human immunoglobulin (PDB_ID: 3SGK and 3SGJ).

### Comparative modeling

We calculated three structural models of a quaternary protein complex of a chimeric antibody against TNF*α* interacting with the Fc*γ*RIIIA receptor by using PyMOL (De Lano 2002) and SwissPDBViewer (Guex and Peitsch 1997). Each glycosylation ladder coming from the crystal structures here investigated (1IGT, 3SGJ, 3SGK) was alternatively retained within the three generated structural models. After superimposition operations, allowing backbone connections, we renumbered all the atoms and the residues present in the resulting three final pdb files, by using an in‐house developed Perl script. The obtained final models were examined in VMD (Humphrey et al. 1996), PyMOL, and SPDBV according to our protocol (Pierri et al. 2010). Notably, where side‐chain packing led to clashes in the protein quaternary structure models, alternative side‐chain rotamers were evaluated.

### Molecular dynamics simulations

In order to evaluate the stability of the built 3D models, we performed molecular dynamics (MD) simulations at 300 K of all‐atom structures of *Chimera 1A* and *Chimera 2* (at neutral pH) by using NAMD2 software (Phillips et al. 2005). For our simulations, we used the CHARMM force field (MacKerell et al. 1998) with cmap correction (Mackerell et al. 2004). In the setup phase, the psfgen tool of VMD (Humphrey et al. 1996) has been used to generate a complete all‐atom psf file of the system.

Each starting structure was solvated in a TIP3P water rectangular box (*Chimera 1A*, box dimensions 118 × 168 × 165; *Chimera 2*, box dimensions 187 × 167 × 97) via Tcl scripting using a padding of 15 Å. Periodic boundary conditions and Particle Mesh Ewald (PME) were applied for full electrostatic calculations. The *Chimera 1A* all‐atom system was constituted of 296,802 atoms, while the *Chimera 2* all‐atom system was constituted of 297,902 atoms. In order to limit the total number of atoms, TNF*α* atoms and carbohydrates moieties were not included in MD runs. “AutoIonize” plug‐in was used to add seven Na^+^ and three Na^+^ counterions to neutralize the two systems, respectively; a protection shell of 5 Å from solute was chosen and a minimum distance of 5 Å between ions was imposed. An even more severe minimization/equilibration protocol (Bossis and Palese 2013; Bossis et al. 2014) was used for solving putative clashes due to the superimposition of protein domains coming from different protein templates. For the initial parts integrator time step was set to 1 fs and “rigidbonds” parameter was imposed to water molecules solely. First, the system underwent 20,000 conjugate gradient minimization steps with backbone harmonically restrained (spring constant *k* = 4.186 KJ/molÅ^2^), followed by a 20,000 conjugate gradient minimization with decreased harmonic constraint (spring constant *k* = 0.251 KJ/molÅ^2^). With the same decreased harmonic constraint, the system was again minimized for another 20,000 steps with conjugate gradient method tuning parameters different from default of “minTinyStep,” “minBabyStep,” and “minLineGoal” (look at NAMD manual for details). Then, a velocity quenching minimization was performed for 40,000 steps, setting maximum move per step at 0.5 Å, with harmonic constraint 0.251 KJ/molÅ^2^. The whole system then underwent another period of 40,000 steps of velocity quenching without restrains. After that the first 60 ps of thermalization at 5 K was performed again with protein backbone restrained at 4.186 KJ/molÅ^2^. The system was again minimized with velocity quenching method for 50,000 steps without constraints. At this point, the temperature of the whole system was increased to 30 K for 50 ps without restrains and then, again, the system was minimized for the last time with conjugate gradient method (and default parameters) for 50,000 steps. Finally, the temperature was increased to 50 K for 50 ps, no constraints were applied. At this level the integrator time step was switched to 2 fs, a frame was saved every 5000 steps (10 ps), “rigidbonds” parameter was set to “all,” and the system was heated according to the following ramp temperature: 100 K for 50 ps, 150 K for 100 ps, 200 K for 200 ps, 250 K for 400 ps, and 300 K for 400 ps. All this preconditioning protocol amounts to 1.55 ns. The production run lasted for further neat 2 ns, concluded the protocol described above.

### Energy calculations

We estimated the free energy of unfolding (deltaG) of *Chimera 2* and *Chimera 1A* by using the FoldX Stability command implemented in the Yasara software (Krieger et al. 2002). This energy estimation represents the difference in free energy between the folded state and the unfolded state of both chimeric antibodies. For each chimeric antibody model the lower the energy, the more stable the structure is (Van Durme et al. 2011). Furthermore, the FoldX AnalyseComplex assay was performed. This command was used to determine the interaction energy between the Fc*γ*RIIIA receptor chain and the closest antibody chain in *Chimera 2* and *Chimera 1A*. The way the FoldX AnalyseComplex operates is by unfolding the selected targets and determining the stability of the remaining molecules and then subtracting the sum of the individual energies from the global energy. More negative energies indicate better binding. Positive energies indicate no binding (Van Durme et al. 2011).

## Results

### Sequence alignment

**Figure 1:**
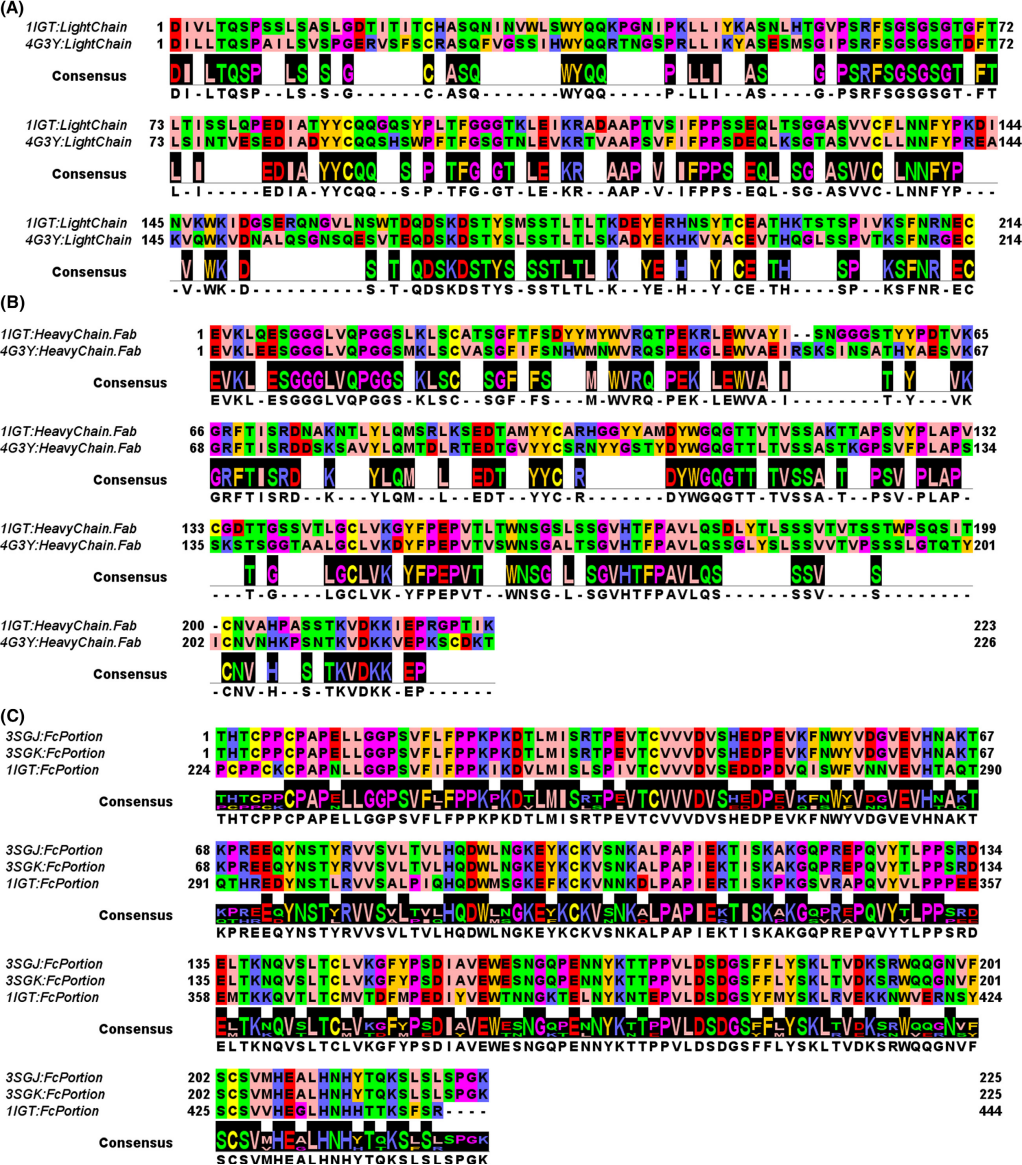
Multiple sequence alignment of antigen‐binding fragment (Fab) and crystallizable fragment (Fc) portions. (A) Pairwise alignment of the light chains from 1IGT (murine IgG) and 4G3Y (human infliximab). (B) Pairwise alignment of the heavy chains of the Fab portions from 1IGT and 4G3Y. (C) Multiple sequence alignment of the Fc portions from 1IGT (murine IgG), 3SGJ (murine Fc portion from crystals grown in the presence of fucose), and 3SGK (murine Fc portion, from crystals grown in the absence of fucose).

We aligned the sequences of the light chains of the Fab fragment of the infliximab (PDB_ID: 4G3Y) (Liang et al. 2013) with the light chains of the Fab fragment of a murine immunoglobulin (PDB_ID: 1IGT) (Harris et al. 1997). Both sequences are long 214 amino acids and we calculated the 58.41% of identical amino acids among the light chains of the two aligned Fab fragments (Fig. 1A). We then aligned the sequences of the heavy chains of the Fab fragment of the infliximab (PDB_ID: 4G3Y) with the heavy chains of the Fab fragment (Fig. 1B) of a murine immunoglobulin (PDB_ID: 1IGT) (Harris et al. 1997). The murine Ig heavy chain (from 1IGT) is 223 amino acids long, whereas the infliximab heavy chain (from 4G3Y) is 226 amino acids long. We calculated the 62.83% of identical amino acids among the heavy chains of the two aligned Fab fragments.

Furthermore, we aligned the sequence of a human IgG–Fc glycoform (PDB_ID: 3SGJ) with the Fc portion (Fig. 1C) of the murine Ig cited above (PDB_ID: 1IGT). The Fc portions of the two Ig (1IGT, 3SGJ/3SGK) were both 225 amino acids long. We calculated the 64.71% of identical amino acids among the Fc portions of the aligned Fc domains.

### Comparative modeling

**Figure 2:**
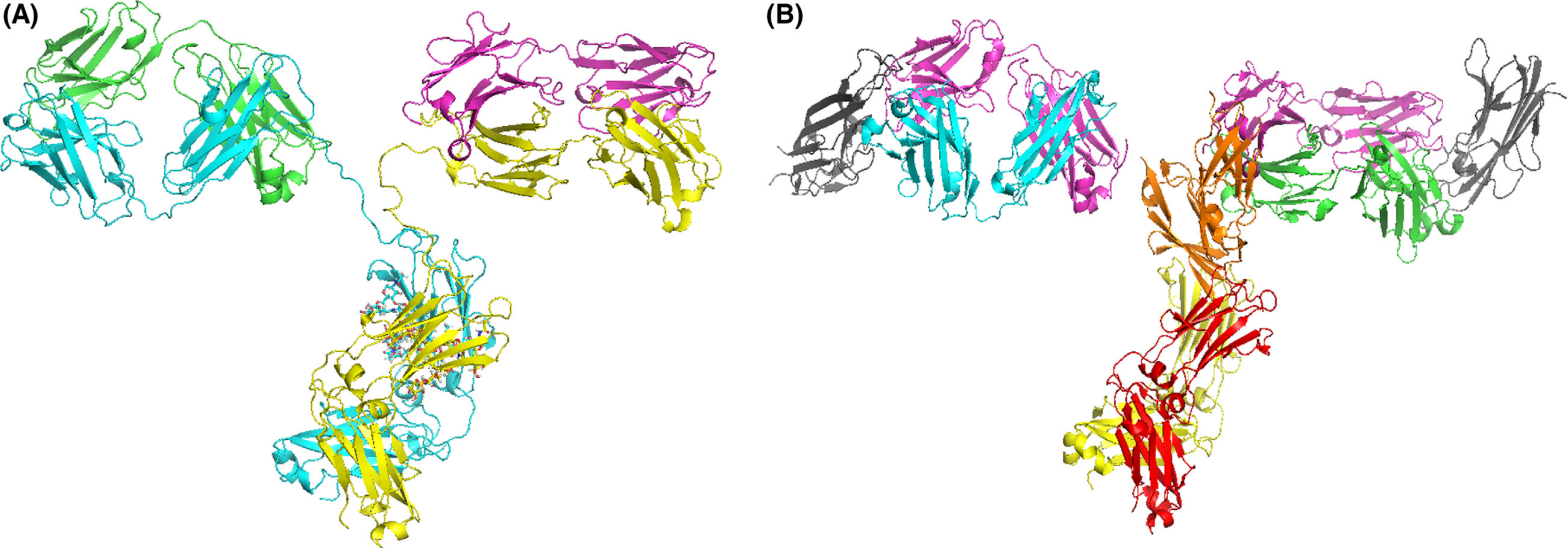
(A) The overall structure of 1IGT antibody used as a protein template for building our chimeric mAb models. The heavy chains are reported in cyan and yellow cartoons, and the light chains are reported in magenta and green cartoons. (B) mAb fragments from 4G3Y (heavy chains from the antigen‐binding fragment (Fab) are reported in cyan and green cartoons, light chains of the Fab fragments are reported in magenta cartoon, and TNF *α* is reported in gray cartoon) and 3SGK (the heavy chain of the crystallizable fragment [Fc] is reported in yellow and red cartoons and Fc*γ* RIIIA is reported in orange cartoon) to be superimposed on 1IGT for building the chimera models listed in Table 1.

For building our structural chimera models we used the structure of the single Fab fragment of the infliximab crystallized in complex with TNF*α* (4G3Y), the two structures of a human IgG–Fc glycoform complexed with a portion of the human Fc*γ*RIIIA receptor (the afucosylated 3SGK and the fucosylated 3SGJ) (Ferrara et al. 2011), and the complete structure of a murine immunoglobulin (1IGT) used either as a protein template (Harris et al. 1997) to drive the 3D modeling or to extract its Fc portion for building one of our chimera models. First, the infliximab Fab fragment was duplicated by using PyMOL and the two Fab fragments obtained were superimposed on the Fab domains of the 1IGT protein template (Fig. 2).

Given that the IgG–Fc complexed with the human Fc*γ*RIIIA receptor was crystallized in the presence and absence of core fucose (3SGJ and 3SGK, respectively), we superimposed both the IgG–Fc glycoform portions complexed with the human Fc*γ*RIIIA receptor on the Fc fragment of the 1IGT protein template for building two chimeric models for comparative purposes.

At the end of our superimposition operations, we obtained three quaternary structure models of two chimera antibodies. Two chimera mAb models were built in the presence of the two above‐cited Fc fragments and glucose ladders containing fucose extracted from 3SGJ and 1IGT, whereas the last mAb model was built in the presence of the Fc fragment and a glucose ladder afucosylated extracted from 3SGK (Table 1).

**Table 1:** Monoclonal antibody (mAb) portions connected for building the different chimeric antibodies. The composition of 3D chimera models built in this study was performed using different crystal fragments present in the PDB database: 4G3Y.pdb, light and heavy chain human Fabs complexed with TNF*α*; 3SGJ.pdb and 3SGK.pdb, Fc fragment fucosylated and afucosylated from a human immunoglobulin IgG complexed with FcγRIIIA; 1IGT.pdb complete murine IgG2A immunoglobulin

mAb fragment	*Chimera 1A*	*Chimera 1B*	*Chimera 2*
TNF*α* + Fab_s_	4G3Y	4G3Y	4G3Y
Fc_s_	1IGT	3SGJ	3SGK
Fc glucose ladder	1IGT (3SGJ)	3SGJ (1IGT)	3SGK
Fc*γ*RIIIA	3SGJ	3SGJ	3SGK
Fc fucosylation	Yes	Yes	No
Fc glycosylation site (residues numbering from crystals)	294‐EDYNSTLR‐301	72‐EQYNSTYR‐79	72‐EQYNSTYR‐79
Fc glycosylation site (residues numbering from chimeras)	298‐EDYNSTLR‐305	297‐EQYNSTYR‐304	297‐EQYNSTYR‐304
Fab fragment (heavy chain from 4G3Y) to be connected to the Fc fragment	223‐CDKT‐226	223‐CDKT‐226	223‐CDKT‐226
Fc fragment (residues from 1IGT/3SGJ/3SGK) residues to be connected to Fab_s_ (residues numbering from crystals)	224‐PCPPCKC‐230	4‐CPPCPAP‐10	1‐THTCPPCPAP‐10
Fc fragment (residues from 1IGT/3SGJ/3SGK) residues to be connected to Fab_s_ (residues numbering from chimeric models)	227‐PCPPCKC‐233	227‐THTCPPCPAP‐237	227‐THTCPPCPAP‐237

Fc, crystallizable fragment; Fab, antigen‐binding fragment.

Underlined letters indicate the position of the asparagine involved in glycosylation/fucosylation events.

More in detail, the first model called *Chimera 1A* consists of two infliximab–Fab fragments (from 4G3Y) and a human fucosylated IgG–Fc fragment (from 1IGT) complexed with the human glycosylated Fc*γ*RIIIA receptor (from 3SGJ). Two glucose ladders containing *β*‐d‐mannose (BMA), *N*‐acetyl‐glucosamine, *α*‐d‐galactose (GAL), *α*‐d‐mannose (MAN), and *α*‐l‐fucose (FUC) were alternatively retained in *Chimera 1A* from 1IGT.pdb or 3SGJ (see Table 1).

The second model called *Chimera 1B* antibody consisted of two infliximab–Fab fragments (from 4G3Y.pdb) and a human fucosylated IgG–Fc fragment (from 3SGJ) complexed with the human glycosylated Fc*γ*RIIIA receptor (from 3SGJ). Also for *Chimera 1B*, both glucose ladders from 1IGT or 3SGJ were alternatively retained (see Table 1).

The 3D model called *Chimera 2* consisted of two infliximab–Fab fragments (from 4G3Y) and a human IgG–Fc fragment (from the afucosylated 3SGK) complexed with the human Fc*γ*RIIIA receptor (from 3SGK). The glucose ladder containing *N*‐acetyl‐glucosamine, *α*‐d‐mannose, and *α*‐d‐mannose without fucose was retained from 3SGK.

According to our complete sequence alignment (Fig. S1) for building our chimeric antibodies, we directly connected the C‐terminal of the infliximab Fab portion (223‐CDKT‐226 from 4G3Y) to the N‐terminal of the Fc portion (224‐PCPPCKC‐230 from 1IGT or 1‐THTCPPCPAP‐10 from 3SGK), whereas we added the tripeptide “227‐THT‐229” to the C‐terminal of the infliximab (i.e., 223‐CDKT‐226 from 4G3Y) before connecting it to the N‐terminal of the Fc portion from the human immunoglobulin (4‐PCPPCKC‐10, from 3SGJ), lacking the initial THT tripeptide.

### Crucial interactions between the Fc fragment and the Fc*γ*RIIIA receptor

**Figure 3:**
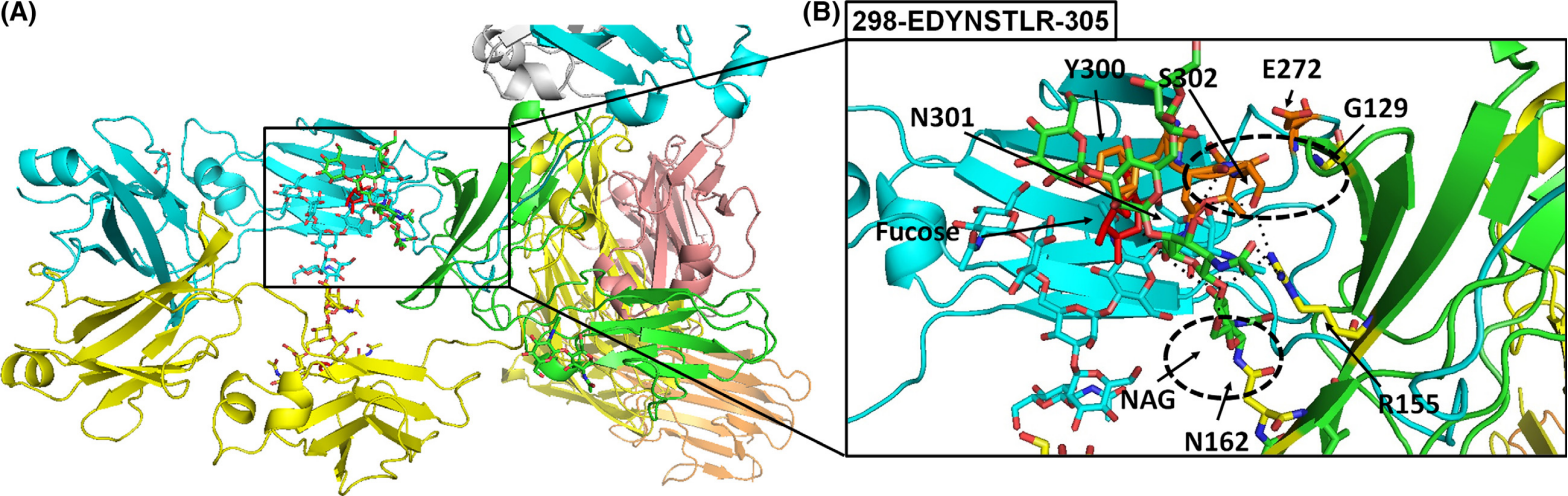
3D overall structures of the *Chimera 1A* antibody. (A) Top view is the structure of the fucosylated *Chimera 1A* mAb. The crystallizable fragment (Fc) chain (fucosylated) is shown in cyan yellow. The receptor is shown in green. The oligosaccharides are depicted as sticks representations and fucose is reported in red sticks. (B) Exploded view on the interaction interface between fucosylated and Fc receptor and Fc*γ* RIIIA. Interatomic distances below the 4 Å are highlighted by black dashed lines by using the distance wizard implemented in PyMOL.

**Figure 4:**
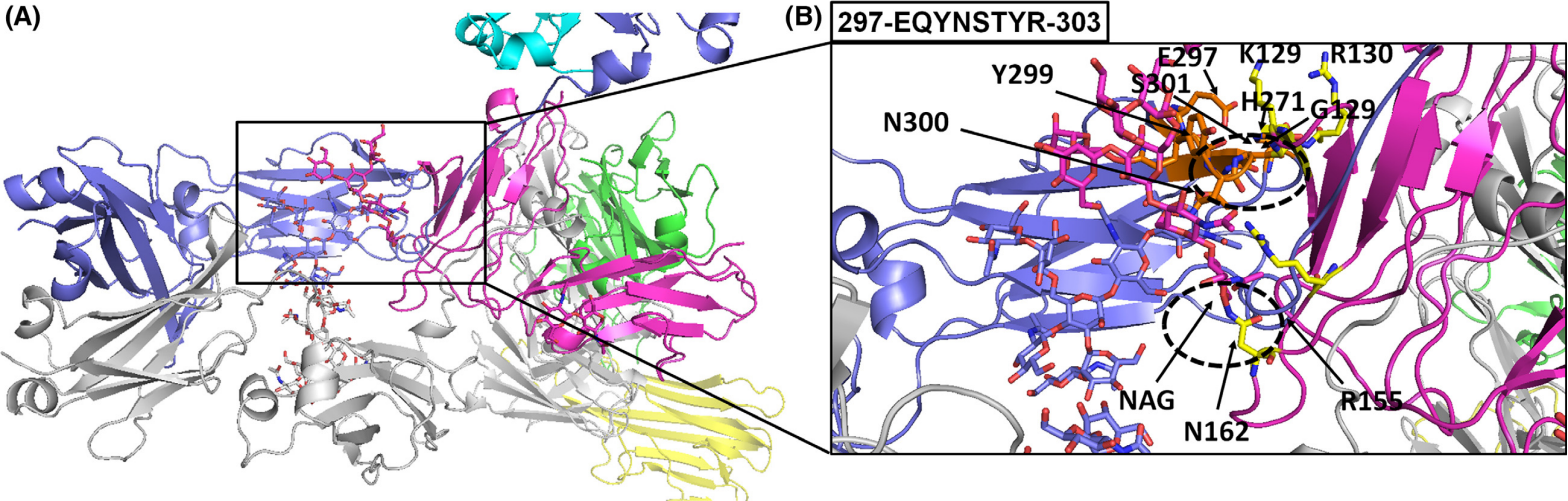
3D overall structures of the *Chimera 2* antibody. (A) Top view is the structures of the afucosylated *Chimera 2* mAb. The crystallizable fragment (Fc) chain (afucosylated) is shown in blue white. The receptor is shown in magenta. The oligosaccharides are depicted as sticks representations. (B) Exploded view on the interaction interface between afucosylated crystallizable fragment (Fc) receptor and Fc*γ* RIIIa. Interatomic distances below the 4 Å are highlighted by black dashed lines by using the distance wizard implemented in PyMOL.

Interactions among the Fc portions from 3SGK and 3SGJ (forming the Fc portions of *Chimera 2* and *Chimera 1B*, respectively) and Fc*γ*RIIIA (cocrystallized within 3SGK and 3SGJ) in the absence/presence of fucose, respectively, have been already described (Ferrara et al. 2011). Furthermore, we noticed that *Chimera 1A* and *Chimera 1B* share very similar features from a structural point of view after their energy minimization (the static rmsd between the two chimera backbones was about 2 Å with both glycosylation ladders alternatively retained from 1IGT or 3SGJ), whereas the main differences were observed in their comparison with *Chimera 2*. Therefore, we focused our attention and following analyses on the estimation of interactions among Fc portions in the context of our chimera (energetically minimized) antibodies *Chimera 2* and *Chimera 1A* (in particular, *Chimera 1A* with the glycosylation ladder retained from 3SGJ showing fucose interacting with the glycosylation ladder of the Fc*γ*IIIRA receptor). It is known that fucosylation of the residue Asn297/300 (N300 in *Chimera 2* and N301 in *Chimera 1A*) can decrease the affinity of the mAb Fc portion for the Fc*γ*RIIIA. On the basis of this assumption, we highlighted residues of the loop containing the specific Asn glycosylation site in our two chimeric models according to our multiple sequence alignment (Fig. 3, Tables 1 and 2). The Asn glycosylation site containing loop within our *Chimera 1A* model consists of residues 298‐EDYNSTLR‐305, whereas the Asn glycosylation site containing loop within our *Chimera 2* model consists of residues 297‐EQYNSTYR‐304 (Fig. 4, Tables 1 and 3). We selected residues within 4 Å from those loops in order to understand how the presence of fucose can contribute to change the network of bonds and interactions among those loop residues and closest residues (amino acids and carbohydrate units) of the Fc*γ*RIIIAfragment.

**Table 2:** Crucial interactions between the *Chimera 2* mAb model in the absence of fucose and Fc*γ*RIIIA receptor

*Chimera 2* residues of the mAb binding loop 297‐EQYNSTYR‐304	Residues within 4 Å from the Fc mAb binding loop 297‐EQYNSTYR‐304	Fc*γ*RIIIA amino acids and glucose ladder carbohydrate units within 4 Å from the Fc mAb binding loop 297‐EQYNSTYR‐304	Fc glucose ladder carbohydrate units within 4 Å from the Fc mAb binding loop 297‐EQYNSTYR‐304
E297	H271 – E296 – Q298 S301 – T302 – Y303		
Q298	E296 – E297 – Y299 – N300 – S301 – T302 – R304	NAG1445 – NAG1447	MAN1472
Y299	Q298 – N300 – S301		BMA1470 – MAN 1475 – MAN 1477 **K128 – G129**
N300	Q298 – Y299 – S301 – T302	NAG1445	NAG1445 – NAG1447 **G129 – R155**
S301	H271 – E297 – Q298 – Y299 – N300 – T302		**K128 – G129 – R130**
T302	L237 – V269 – E296 – E297 – Q298 – N300 – S301 – Y303	NAG1445	
Y303	V266 – D268 – V269 – H271 – P274 – R295 – E296 – E297 – T302 – R304		
R304	V265 – V266 – V267 – R295 – E296 – E298 – Y303 – V305 – V306	NAG1447 – NAG1450 – NAG1453	MAN1472

The residues involved in the interaction between *Chimera 2* mAb model and Fc*γ*RIIIA receptor are reported. The *Chimera 2* model built in this study was obtained by using the cited different crystal fragments present in the PDB database.

Bold labels indicate aminoacids involved in strong interactions between the antibody and the receptor in Chimera2.

**Table 3:** Crucial interactions between the *Chimera 1A* mAb model in the presence of fucose and Fc*γ*RIIIA receptor. The residues involved in the interaction between *Chimera 1A* mAb model in the presence of fucose and FcγRIIIA receptor are reported. The *Chimera 1A* model built in this study was obtained by using the cited different crystal fragments present in the PDB database

*Chimera 1A* residues of the Fc mAb binding loop 298‐EDYNSTLR‐305	Residues within 4 Å of interatomic distance from the Fc mAb binding loop 298‐EDYNSTLR‐305	Fc*γ*RIIIA amino acids and glucose ladder carbohydrate units within 4 Å from the Fc mAb binding loop 298‐EDYNSTLR‐305	Fc glucose ladder carbohydrate units within 4 Å from the Fc mAb binding loop 298‐EDYNSTLR‐305
E298	H296 – R297 – D299 – L304 – R305		
D299	E298 – Y300 – T303 – L304		
Y300	D299 – N301 – S302 – T303 – R305	NAG501 – **FUC508**	BMA303 – MAN304
N301	Y300 – S302 – T303	NAG501 – **FUC508**	NAG301 – NAG302 – BMA303
S302	D272 – Y300 – N301 – T303		**G129 – R155 –** BMA303
T303	E272 – D299 – Y300 – N301 – S302 – L304	NAG501	
L304	D269 – V270 – E272 – P275 – E298 – D299 – Y300 – T303 – R305	NAG501	
R305	V266 – V267 – R297 – E298 – D299 – Y300 – L304 – V306	NAG501 – NAG502 – **FUC508**	MAN304

Bold labels indicate the fucose and those aminoacids of Chimera1A that in presence of fucose form interactions weaker than homologous interactions observed in Chimera2.

We observed that in the absence of fucose in the *Chimera 2* model the Fc portion of the mAb was in general closer to the Fc*γ*RIIIA fragment. Notably, some residues of the loop 297‐EQYNSTYR‐304 were reoriented toward the Fc*γ*RIIIA and some strong interactions were established among residues of the Fc loop and residues of the Fc*γ*RIIIA domain. In particular, we observed the formation of strong interactions between S301 of the Fc fragment and G129 of the Fc*γ*RIIIA and we noticed a characteristic motif 270‐SHEDPE‐275 (see *Chimera 2* model, Fig. 3 and Table 2) that forms new ionic interactions with residues of the loop 297‐EQYNSTYR‐304. In particular, an ionic interaction (2.5 Å long) was established between H271 and E297 that most likely also triggers the reorientation of the loop containing residues Y299 and N300. Polar/H‐bond interactions are also observed between residues Y299, N300, and S301 on the Fc fragment and residues G129 (interacting with Y299, N300, and S301), K128 (interacting with Y299 and S301), R130 (interacting with S301), and R155 (interacting with N300). More in general, in the absence of fucose, residues of the loop 297‐EQYNSTYR‐304 appear to be closer to the loop 128‐KGR‐130 on the Fc*γ*RIIIA and less constrained in the space toward Fc*γ*RIIIA, although in the presence of a similar amount of other carbohydrate units (Fig. 4, Table 3).

In *Chimera 1A* in the presence of fucose, G129 of the Fc*γ*RIIIA fragment forms weaker interactions with residues of the Fc fragment. More in general, those interactions are observed among residues of the loop 298‐EDYNSTLR‐305 and residues of the Fc*γ*RIIIA fragment. It was also observed that in correspondence of the H271 residue present in the *Chimera 2* model we have an acidic residue in the *Chimera 1A* model and no direct interactions are detectable between the motif 271‐SEDDPD‐276 and residues of the loop 298‐EDYNSTLR‐305. It appears that E298 is instead involved in local interactions with basic residues H296, R297, and R305 producing a loop protruding toward the carbohydrate (fucose containing) ladder. More, in general, residues of the loop 298‐EDYNSTLR‐305 appear to be more distant from Fc*γ*RIIIA and more constrained in a network of interactions with carbohydrate units (see Fig. 3 and Table 2). In particular, we observed in the presence of fucose direct interactions within 4 Å among fucose and residues Y300, N301, and R305 of the loop 298‐EDYNSTLR‐305. The same residues form stable interactions with NAG501 (all the residues), NAG502 (only R305), NAG301, NAG302 (only N301), and BMA303 (Y300 and N301) and MAN304 (Y300). The presence/absence of fucose in the Fc fragments taken from 1IGT (or 3SGJ) or 3SGK do not affect the strong interaction between N162 and NAG301 in *Chimera 1A* model or the interaction between N162 and NAG1445 in *Chimera 2* also after having minimized the two chimera complexes.

**Figure 5:**
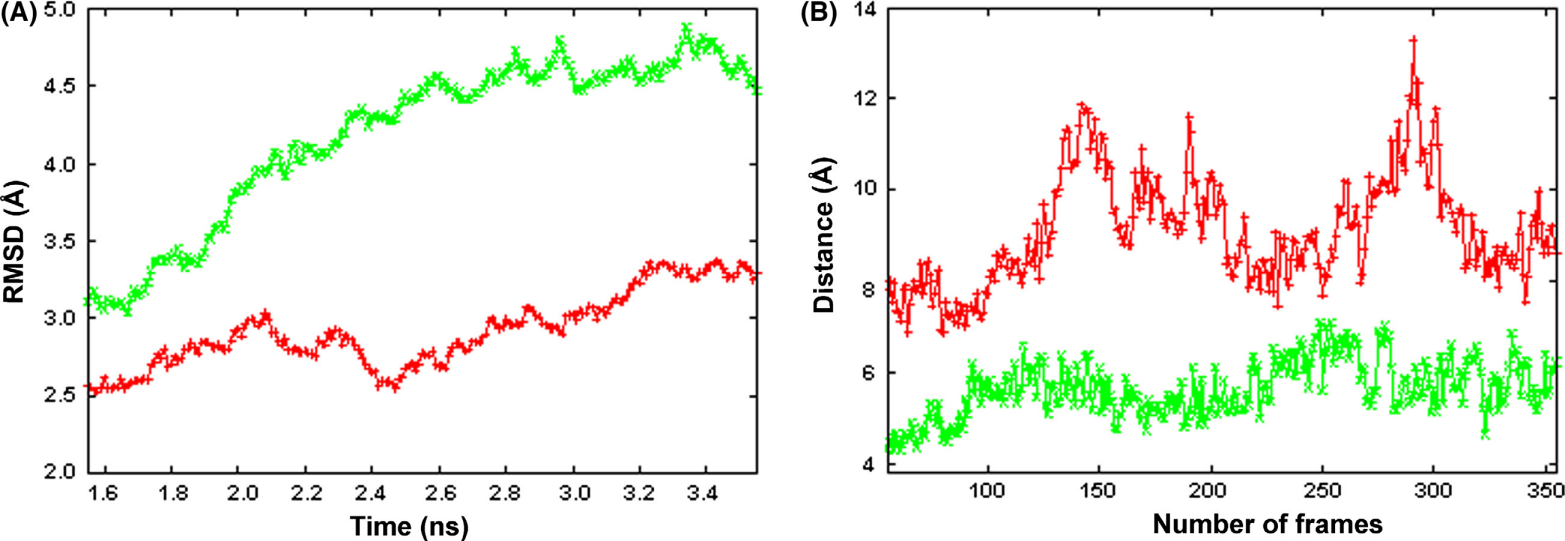
Molecular dynamics (MD) simulations. (A) RMSDs of *Chimera 1A* (red) and *Chimera 2* (green) of the whole protein 3D models (hydrogen atoms are not considered for RMSD calculations) in complex with Fc*γ*RIIIA, during 2 ns production runs. RMSD average values and standard deviations relative to production runs are 2.95 ± 0.32 (*Chimera 1A*, red plot) and 4.28 ± 0.56 (*Chimera 2*, green plot). (B) Distances between the centers of mass of G129 (receptor subunit) and S302 (closest antibody subunit) in *Chimera 1A* model (red) and between the centers of mass of G129 (receptor subunit) and S301 (closest antibody subunit) in *Chimera 2* model (green) versus the number of frames during production runs are reported. This figure was obtained with gnuplot (http://www.gnuplot.info/).

In order to investigate more deeply the stability of interactions occurring between the triplet residues K128‐G129‐R130 and residues of the loops 297‐EQYNSTYR‐304 in *Chimera 2* and 298‐EDYNSTLR‐305 in *Chimera 1A*, we also monitored the distance between the center of mass of the two set of residues and the distance between the center of mass of G129 and S301 (close to the glycosilation site N300) in *Chimera 2* or S302 (close to the glycosilation site in N301) in *Chimera 1A* into two independent 2 ns MD production runs (Fig. 5, panel A). Notably, before MD runs, the distances between the center of mass of G129 (receptor chain) and S301 in *Chimera 2* or S302 in *Chimera 1A* were about 5 and 5.5 Å respectively, whereas, after MD runs, the distance remains further stable only in *Chimera 2* (below 6 Å) while increasing in *Chimera 1A* (between 7 and 11 Å) (Fig. 5, panel B). A minimum distance between residues S301 and G129 in *Chimera 2* is again observed at 3 Å useful for establishing potentially covalent bonds. Also, the free energies of unfolding of both chimeric 3D models and the interaction energies calculated between the receptor chain and the closest antibody chain in *Chimera 2* and *Chimera 1A* confirm a greater stability and a better binding within *Chimera2* 3D complex model (see Table 4).

**Table 4:** Energy calculations on 3D models of *Chimera 1A* and *Chimera 2* before and after molecular dynamics (MD) runs

Free energy of Unfolding (FoldX Stability)				
	*Chimera 1A* before MD runs	*Chimera 2* before MD runs	*Chimera 1A* after 2 ns MD runs	*Chimera 2* after 2 ns MD runs
Total energy (kcal/mol)	759.20	920.57	710.074	296.381
Backbone Hbond	−862.97	−873.78	−752.668	−811.358
Sidechain Hbond	−243.51	−259.01	−273.993	−445.516
Van der Waals	−1691.34	−1710.85	−1560.22	−1520.93
Electrostatics	−56.90	−58.59	−83.3617	−66.8915
Solvation polar	2357.03	2394.64	2170.46	2015.5
Solvation hydrophobic	−2159.72	−2186.73	−1996.63	−1977.47
Van der Waals clashes	274.48	485.84	129.53	45.4159
Entropy side chain	818.76	828.27	809.921	853.432
Entropy main chain	2317.25	2258.53	2237.97	2205.79
*cis*_bond	17.21	19.10	18.2409	16.9042
Torsional clash	49.11	82.16	71.7265	43.0954
Backbone clash	404.81	496.37	363.938	345.245
Helix dipole	−0.88	−2.84	−2.59994	−6.1477
Disulfide	−60.50	−59.23	−58.1953	−57.014
Electrostatic kon	−3.87	−2.07	−3.86614	−3.01727
Energy ionization	5.06	5.13	3.74828	4.58387

Interaction energies (FoldX AnalyseComplex)				
	*Chimera 1A* before MD runs	*Chimera 2* before MD runs	*Chimera 1A* after 2 ns MD runs	*Chimera 2* after 2 ns MD runs
Group 1	S (receptor)	S (receptor)	S (receptor)	S (receptor)
Group 2	B (closest antibody chain)	Z (closest antibody chain)	B (closest antibody chain)	Z (closest antibody chain)
Intraclashes Group 1	34.57	93.00	22.05	8.14
Intraclashes Group 2	42.32	60.05	56.12	24.45
Interaction energy (kcal/mol)	82.95	134.88	−3.96	−9.11

## Discussion

Comparative modeling is generally used to build 3D models of proteins and enzymes whose crystal structure is not yet available (Pierri et al. 2010). Lot of proteins can be modeled as long as a good 3D protein template is available (Pierri et al. 2010). For what concerns antibodies, molecular modeling was mainly used for improving the antibody–antigen affinity by mutagenesis of antibody complementary determining regions (CDR) through in silico mutagenesis and the following assays on the bench (Hou et al. 2008). Notably, thanks to the crystallization of three complete antibodies, it is now possible to build a complete mAb model based on the available three mAb templates 1IGT (Harris et al. 1997), 1IGY (Harris et al. 1998), 1HZH (Saphire et al. 2001) for investigating both antibody–antigen interactions and antibody–receptor interactions. In particular, here we investigated the influence of the glycosylation/fucosylation on the interactions between the Fc*γ*RIIIA and the Fc fragments within the two in‐house developed chimera mAb models *Chimera 1A* and *Chimera 2*. In order to validate our mAb modeling project, before proceeding with the 3D comparative modeling, we aligned the sequences of the light chains of the Fab fragment of the infliximab (4G3Y) (Liang et al. 2013) with the light chains of the Fab fragment of the murine immunoglobulin 1IGT (Harris et al. 1997). Both sequences are long 214 amino acids and share the 58.41% of identical amino acids. Then, we aligned the sequences of the heavy chains of the Fab fragment of the infliximab (4G3Y) (Liang et al. 2013) with the heavy chains of the Fab fragment of the murine immunoglobulin 1IGT (Harris et al. 1997). The murine Ig heavy chain (from 1IGT) is 223 amino acids long, whereas the infliximab heavy chain (from 4G3Y) is 226 amino acids long. The two heavy chains share the 62.83% of identical amino acids. Finally, we aligned the sequence of the human IgG–Fc glycoform (3SGJ or 3SGK) (Ferrara et al. 2011) with the Fc portion of the murine Ig (1IGT.pdb) (Harris et al. 1997). The Fc portions of the two Ig (1IGT, 3SGK) were both 225 amino acids long and share the 64.71% of identical amino acids. For building the two chimera mAbs we used comparative modeling techniques (Pierri et al. 2010).

We observed that in the absence of fucosylation within *Chimera 2* the Fc and Fc*γ*RIIIA are quite closer and in particular new strong interactions are established between G129 (Fc*γ*RIIIA) and S301 (Fc mAb fragment) as well as new polar interactions are established between Y299, N300, and S301 from the Fc mAb fragment and residues K128, G129, R130, and R155 from the Fc*γ*RIIIA receptor. Each chimeric 3D model complex was also minimized and relaxed in two independent 2 ns MD production runs. Notably, *Chimera 2* 3D complex model appears more stable after MD simulations than *Chimera 1A* according to what observed by measuring the free energies of unfolding of both chimeric 3D models. Furthermore, the calculated interaction energies confirm that interactions of *Chimera 2* with Fc*γ*RIIIA are stronger than those observed between *Chimera 1A* and Fc*γ*RIIIA.

The IgG1Fc–Fc receptor interactions may play a critical role in determining the biological activities of the therapeutic mAbs. The bivalent mAbs containing the FAB and Fc fragments such as infliximab, adalimumab, and golimumab show a large spectrum of biological activities. Golimumab, however, shows a minor apoptosis activity as compared to adalimumab and infliximab (Ueda et al. 2013; Urbano et al. 2014). Both adalimumab and infliximab induce apoptosis in cultured monocytes. Apoptosis induction was caspase‐dependent and detectable already after 2 h. The production of interleukin‐10 and interleukin‐12 by monocytes was downregulated significantly by adalimumab and infliximab but not by etanercept, while levels of soluble tumor necrosis factor in monocyte cultures were downregulated by all three monoclonal antibodies indicating that all three mAbs interact with the soluble TNF*α*, but only adalimumab and infliximab are able to neutralize the actions of the transmembrane TNF*α* (Shen et al. 2005).

In contrast, the monovalent mAb certolizumab pegol (lacking the Fc fragment) lost the CDC and ADCC activities; etanercept, showing weak Fc–Fc receptor interactions, demonstrated a reduced ADCC (Arora et al. 2009; Kaymakcalan et al. 2009). Certolizumab and etanercept also demonstrated a reduced clinical efficacy as compared to infliximab and adalimumab in the treatment of inflammatory bowel diseases (IBD), thereby suggesting that the CDC and ADCC activity of the mAbs may play a role in the IBD. Given that the efficacy in IBD diseases beyond Fab–TNF*α* interactions may also depend on the ADCC‐related Fc and Fc*γ*RIIIA interactions, we would expect that our *Chimera 2* should be more efficient than *Chimera 1A* in treating inflammatory TNF*α*‐related diseases including IBD.

In our chimeric mAbs, we observed that the fucosylation of the Fc fragment can reduce the number and impair the quality of interactions of the carbohydrate–carbohydrate interface between Fc*γ*RIIIA glycans and the (fucosylated/afucosylated) Fc fragment, without affecting the TNF*α*–Fab interactions. In the fucosylated complex these interactions are weakened or nonexistent, if compared with the afucosylated Fc–Fc*γ*RIIIA, explaining the reduced ADCC activity, also described for the infliximab biosimilars and other fucosylated mAbs described previously (Shields et al. 2002; Shinkawa et al. 2003; Ferrara et al. 2011; EMAIAR, 2013; EMARAR, 2013; Feagan et al. 2014; Kapur et al. 2014). This may have impact on the evaluation of therapeutic equivalence of anti‐TNF antibodies.

It should be finally stressed that the availability of a 3D mAb model would help in planning chemical modifications of the different mAb fragments for increasing both mAb(Fab)–antigen affinity, by performing in silico and in vitro mutagenesis of CDR, and mAb(Fc)–receptor affinity, by performing in silico and in vitro mutagenesis of the Fc loop residues, proposed to be involved in direct interactions with Fc*γ*RIIIA.

## Author Contributions

Pierri, Tricarico, and Cetrone participated in research design. Punzi, Bossis, and De Grassi conducted experiments. De Grassi, Bossis, Parisi, and Punzi performed data analysis. Pierri and Tricarico wrote or contributed to the writing of the manuscript.

## Disclosures

The authors declare that there are no conflicts of interest.

## Supporting information


**Figure S1.** Multiple full‐length sequence alignment of 3D crsystallized antibody sequences used for generating 3D models of Chimera2, Chimera1A and Chimera1B.

## References

[prp2197-bib-0001] Arora T , et al. (2009). Differences in binding and effector functions between classes of TNF antagonists. Cytokine 45: 124–131. 19128982 10.1016/j.cyto.2008.11.008

[prp2197-bib-0002] Bossis F , Palese LL (2013). Amyloid beta(1‐42) in aqueous environments: effects of ionic strength and E22Q (Dutch) mutation. Biochim Biophys Acta 1834: 2486–2493. 24016775 10.1016/j.bbapap.2013.08.010

[prp2197-bib-0003] Bossis F , De Grassi A , Palese LL , Pierri CL (2014). Prediction of high‐ and low‐affinity quinol‐analogue‐binding sites in the aa3 and bo3 terminal oxidases from *Bacillus subtilis* and *Escherichia coli* . Biochem J 461: 305–314. 24779955 10.1042/BJ20140082

[prp2197-bib-0004] Curci A , Mele A , Camerino GM , Dinardo MM , Tricarico D (2014). The large conductance Ca(2+) ‐activated K(+) (BKCa) channel regulates cell proliferation in SH‐SY5Y neuroblastoma cells by activating the staurosporine‐sensitive protein kinases. Front Physiol 5: 476. 25538629 10.3389/fphys.2014.00476 PMC4260485

[prp2197-bib-0005] Davies J , et al. (2001). Expression of GnTIII in a recombinant anti‐CD20 CHO production cell line: expression of antibodies with altered glycoforms leads to an increase in ADCC through higher affinity for FC gamma RIII. Biotechnol Bioeng 74: 288–294. 11410853

[prp2197-bib-0006] De Lano WL (2002). Available at http://www.pymol.org/.

[prp2197-bib-0007] EMAIAR (2013). Committee for Medicinal Products for Human Use, “EMA/CHMP/589422/2013”.

[prp2197-bib-0008] EMARAR (2013). Committee for medicinal products for human use, “EMA/CHMP/589317/2013”.

[prp2197-bib-0009] Feagan BG , et al. (2014). The challenge of indication extrapolation for infliximab biosimilars. Biologicals 42: 177–183. 24962198 10.1016/j.biologicals.2014.05.005

[prp2197-bib-0010] Ferrara C , et al. (2011). Unique carbohydrate‐carbohydrate interactions are required for high affinity binding between FcgammaRIII and antibodies lacking core fucose. Proc Natl Acad Sci USA 108: 12669–12674. 21768335 10.1073/pnas.1108455108 PMC3150898

[prp2197-bib-0011] Geremia A , Biancheri P , Allan P , Corazza GR , Di Sabatino A (2014). Innate and adaptive immunity in inflammatory bowel disease. Autoimmun Rev 13: 3–10. 23774107 10.1016/j.autrev.2013.06.004

[prp2197-bib-0012] Guex N , Peitsch M (1997). SWISS‐MODEL and the Swiss‐PdbViewer: an environment for comparative protein modeling. Electrophoresis 18: 2714–2723. 9504803 10.1002/elps.1150181505

[prp2197-bib-0013] Harris LJ , Larson SB , Hasel KW , McPherson A (1997). Refined structure of an intact IgG2a monoclonal antibody. Biochemistry 36: 1581–1597. 9048542 10.1021/bi962514+

[prp2197-bib-0014] Harris LJ , Skaletsky E , McPherson A (1998). Crystallographic structure of an intact IgG1 monoclonal antibody. J Mol Biol 275: 861–872. 9480774 10.1006/jmbi.1997.1508

[prp2197-bib-0015] Hou S , et al. (2008). Humanization of an anti‐CD34 monoclonal antibody by complementarity‐determining region grafting based on computer‐assisted molecular modelling. J Biochem 144: 115–120. 18424812 10.1093/jb/mvn052

[prp2197-bib-0016] Humphrey W , Dalke A , Schulten K (1996). VMD: visual molecular dynamics. J Mol Graph 14: 33–38, 27–38. 8744570 10.1016/0263-7855(96)00018-5

[prp2197-bib-0017] Kapur R , et al. (2014). A prominent lack of IgG1‐Fc fucosylation of platelet alloantibodies in pregnancy. Blood 123: 471–480. 24243971 10.1182/blood-2013-09-527978 PMC3901064

[prp2197-bib-0018] Kaymakcalan Z , et al. (2009). Comparisons of affinities, avidities, and complement activation of adalimumab, infliximab, and etanercept in binding to soluble and membrane tumor necrosis factor. Clin Immunol 131: 308–316. 19188093 10.1016/j.clim.2009.01.002

[prp2197-bib-0019] Kim HY , Kim S , Chung DH (2006). FcgammaRIII engagement provides activating signals to NKT cells in antibody‐induced joint inflammation. J Clin Invest 116: 2484–2492. 16917543 10.1172/JCI27219 PMC1550276

[prp2197-bib-0020] Koshy S , et al. (2013). Blocking KCa3.1 channels increases tumor cell killing by a subpopulation of human natural killer lymphocytes. PLoS ONE 8: e76740. 24146918 10.1371/journal.pone.0076740 PMC3795664

[prp2197-bib-0021] Krieger E , Koraimann G , Vriend G (2002). Increasing the precision of comparative models with YASARA NOVA–a self‐parameterizing force field. Proteins 47: 393–402. 11948792 10.1002/prot.10104

[prp2197-bib-0022] Liang S , Dai J , Hou S , Su L , Zhang D , Guo H , Hu S , Wang H , Rao Z , Guo Y , Lou Z (2013). Structural basis for treating tumor necrosis factor *α* (TNF*α*)‐associated diseases with the therapeutic antibody infliximab. J Biol Chem 288(19): 13799–807. doi: 10.1074/jbc.M112.433961. 23504311 10.1074/jbc.M112.433961 PMC3650416

[prp2197-bib-0023] MacKerell AD , et al. (1998). All‐atom empirical potential for molecular modeling and dynamics studies of proteins. J Phys Chem B 102: 3586–3616. 24889800 10.1021/jp973084f

[prp2197-bib-0024] Mackerell AD , Feig M , Brooks CL (2004). Extending the treatment of backbone energetics in protein force fields: limitations of gas‐phase quantum mechanics in reproducing protein conformational distributions in molecular dynamics simulations. J Comput Chem 25: 1400–1415. 15185334 10.1002/jcc.20065

[prp2197-bib-0025] Nimmerjahn F , Ravetch JV (2008). Fcgamma receptors as regulators of immune responses. Nat Rev Immunol 8: 34–47. 18064051 10.1038/nri2206

[prp2197-bib-0026] Parekh BS , et al. (2012). Development and validation of an antibody‐dependent cell‐mediated cytotoxicity‐reporter gene assay. MAbs 4: 310–318. 22531445 10.4161/mabs.19873 PMC3355484

[prp2197-bib-0027] Phillips JC , et al. (2005). Scalable molecular dynamics with NAMD. J Comput Chem 26: 1781–1802. 16222654 10.1002/jcc.20289 PMC2486339

[prp2197-bib-0028] Pierri CL , Parisi G , Porcelli V (2010). Computational approaches for protein function prediction: a combined strategy from multiple sequence alignment to molecular docking‐based virtual screening. Biochim Biophys Acta 1804: 1695–1712. 20433957 10.1016/j.bbapap.2010.04.008

[prp2197-bib-0029] Saphire EO , et al. (2001). Crystal structure of a neutralizing human IGG against HIV‐1: a template for vaccine design. Science 293: 1155–1159. 11498595 10.1126/science.1061692

[prp2197-bib-0030] Shen C , et al. (2005). Adalimumab induces apoptosis of human monocytes: a comparative study with infliximab and etanercept. Aliment Pharmacol Ther 21: 251–258. 15691299 10.1111/j.1365-2036.2005.02309.x

[prp2197-bib-0031] Shields RL , et al. (2002). Lack of fucose on human IgG1 N‐linked oligosaccharide improves binding to human Fcgamma RIII and antibody‐dependent cellular toxicity. J Biol Chem 277: 26733–26740. 11986321 10.1074/jbc.M202069200

[prp2197-bib-0032] Shinkawa T , et al. (2003). The absence of fucose but not the presence of galactose or bisecting N‐acetylglucosamine of human IgG1 complex‐type oligosaccharides shows the critical role of enhancing antibody‐dependent cellular cytotoxicity. J Biol Chem 278: 3466–3473. 12427744 10.1074/jbc.M210665200

[prp2197-bib-0033] Sondermann P , Huber R , Oosthuizen V , Jacob U (2000). The 3.2‐A crystal structure of the human IgG1 Fc fragment‐Fc gammaRIII complex. Nature 406: 267–273. 10917521 10.1038/35018508

[prp2197-bib-0034] Ueda N , et al. (2013). The cytotoxic effects of certolizumab pegol and golimumab mediated by transmembrane tumor necrosis factor *α* . Inflamm Bowel Dis 19: 1224–1231. 23619715 10.1097/MIB.0b013e318280b169

[prp2197-bib-0035] Urbano PC , Soccol VT , Azevedo VF (2014). Apoptosis and the FLIP and NF‐kappa B proteins as pharmacodynamic criteria for biosimilar TNF‐alpha antagonists. Biologics 8: 211–220. 25114503 10.2147/BTT.S57253 PMC4124053

[prp2197-bib-0036] Van Durme J , et al. (2011). A graphical interface for the FoldX forcefield. Bioinformatics 27: 1711–1712. 21505037 10.1093/bioinformatics/btr254

[prp2197-bib-0037] Zhou Q , et al. (2008). Development of a simple and rapid method for producing non‐fucosylated oligomannose containing antibodies with increased effector function. Biotechnol Bioeng 99: 652–665. 17680659 10.1002/bit.21598

