# Supplementary figures and images for: Molecular modeling of antibodies for the treatment of TNF α‐related immunological diseases
Source: Pharmacol Res Perspect. 2016 Jan 15;4(1):e00197. doi: 10.1002/prp2.197 (PMC4777268; doi:10.1002/prp2.197)

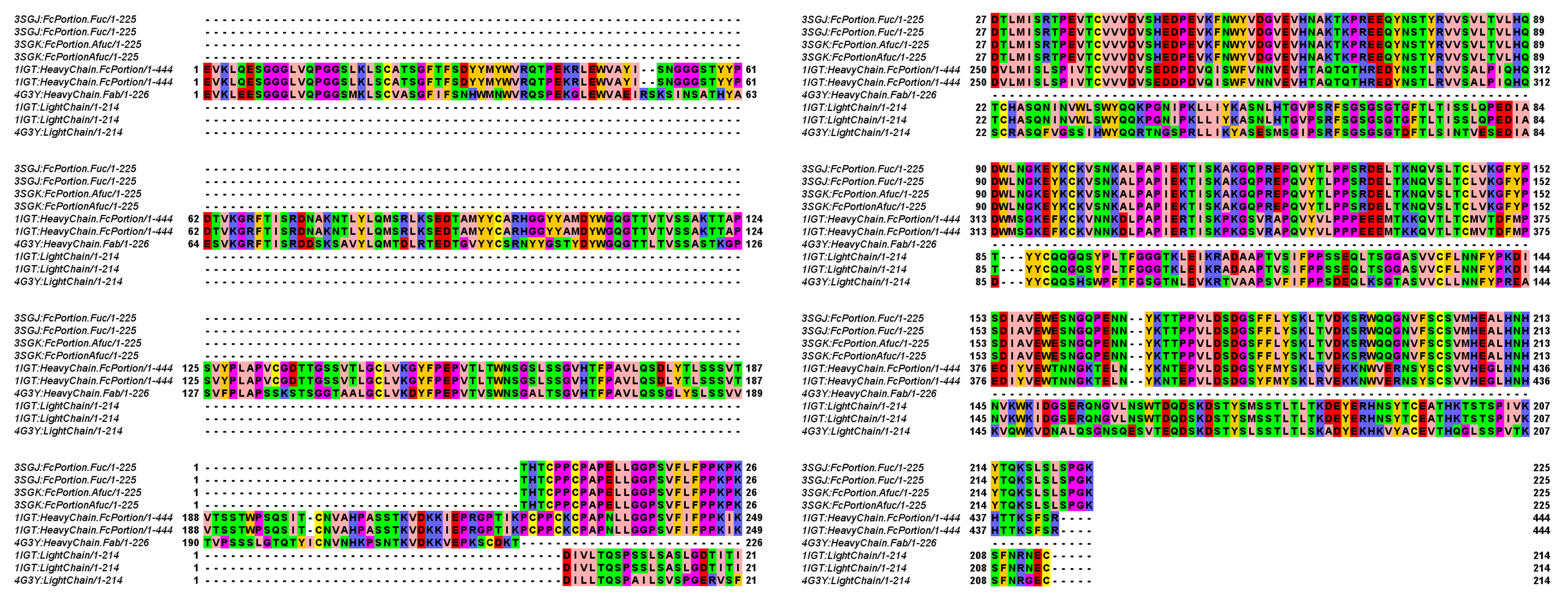

Supplement: Supplementary file 1 — Figure S1. Multiple full‐length sequence alignment of 3D crsystallized antibody sequences used for generating 3D models of Chimera2, Chimera1A and Chimera1B. [file PRP2-4-e00197-s001.jpg]
